# The National Hospital

**Published:** 1901-06-29

**Authors:** 


### The Hospital

A Journal of

The Medical Sciences and Hospital Administration.

Vol. XXX.—No. 770.] Edited by Sir Henry Burdett, K.C.B., and Solomon C. Smith, M.D., M.R.C.P. [June 29, 1901.

### THE NATIONAL HOSPITAL.

The writer of an article on "Provincial Medical Charities," which appeared several years ago in the *Cornhill Magazine*, and excited some attention at the time of its appearance, explained one of the difficulties which then stood in the way of much-needed reforms by saying that it would hardly be possible for a body of English gentlemen to establish a condition on behalf of which nothing could be said. This achievement, however, if we may judge from the report of the committee appointed to inquire into the affairs of the National Hospital for the Paralysed and Epileptic, has been perfectly accomplished by the Board of Management of that institution. This board, we are informed, consisted of twelve elected and of an unspecified number of *ex officio* members; and was supposed to meet monthly, the average number of members present at a meeting being five. There was also a so-called House Committee, consisting of all the members of the board, which was supposed to meet fortnightly, but which actually met only twenty-nine times in two years, while at eighteen of these so-called "meetings" only a single member attended. One member constituted a "quorum" of the Finance Committee, and no record was kept of its proceedings. How the board was originally selected or appointed does not appear, nor is it stated whether the appointments to it were intended to be for life; but we are informed that membership was forfeited by non-attendance for a whole year, and that the members who thus fell off were habitually re-elected to the positions which had been vacated by their neglect. When the discussion about the affairs of the hospital commenced many people found it difficult to understand the often-repeated statement that the addition of two representatives of the medical staff to a board of twelve persons would enable the representatives in question to override the rest of the board, and completely to control the affairs of the institution. The statement is now intelligible. The sole qualification of a large proportion of the ordinary members of the board was that they never entered the doors of the hospital; and it is obvious that, with an "average" attendance of five persons only, the addition of two who constantly attended might easily bring about a re-arrangement of the balance of power. A somewhat ludicrous illustration of the acts of a "meeting" consisting of one person was afforded in the course of the inquiry. A former resident official had given evidence, and Mr. Boydell Haughton, the counsel representing the "Board," rose to cross-examine him for the purpose of diminishing the effect of what he had said. The witness was first asked whether he had not been officially censured by the board, and at once admitted the soft impeachment; but he was permitted by the Committee of Inquiry to explain that he had been censured for something which he had not done, and without either being heard in his defence or even informed that he was accused of anything, the communication of the "censure" being the first that was made to him on the subject. The "acting chairman" of the board promptly admitted that this was not fair treatment, but was unable either to recall the incident or to afford any explanation of it, and thought it hardly possible that he could have been at the "meeting." Being requested to refresh his memory by reference to the minutes, he said in an embarrassed way that he found he had been present, but that the circumstance had wholly escaped his recollection. Asked what other members of the board had attended on the occasion, he was constrained to reply, "There seems to have been no one else."

It is difficult to understand by what form of casuistry the members of this so-called "board" were able to excuse to their own consciences the shameless neglect of duty which has been brought home to them; and it is impossible to be other than curious as to whether the habitually non-attending and constantly re-elected members were ever informed of their re-election or ever wrote letters accepting the responsibilities which it entailed. Questions of this sort, however, will soon fall into the domain of ancient history, and the only important one which can arise out of them will be with reference to the manner in which the government of the hospital shall be conducted for the future. Another meeting of the governors will shortly be called to consider the report, and will probably be well attended; but no meeting of this sort can attempt to enter upon the details of a scheme, and these, of necessity, must be referred to a committee. The best system of hospital management is, we believe, one which provides for a due representation of all interests and encourages personal work. A quarterly court openly elected at the annual meeting may properly decide more important questions, but the actual management of the hospital must be undertaken by a weekly board which may well consist of certain *ex officio* members, such as the chairman, the treasurer, and the honorary secretaries, a certain number of governors, a certain number of representative governors (where any large amount of income is derived from workmen's subscriptions), and a certain number of honorary medical officers elected by the acting staff. The exact proportion of the different classes may vary in different institutions, but the whole board should be a small one. Such a board should embrace every interest; it should be in immediate touch with the paid officers; and where from the size of the hospital it is necessary that there should be one supreme executive officer to carry on the work between the meetings of the board, that officer should be a permanent resident medical officer who would be equally in touch with the medical and the non-medical departments.

